# Study protocol of KLIMOP: a cohort study on the wellbeing of older cancer patients in Belgium and the Netherlands

**DOI:** 10.1186/1471-2458-11-825

**Published:** 2011-10-25

**Authors:** Laura Deckx, Doris van Abbema, Katherine Nelissen, Liesbeth Daniels, Piet Stinissen, Paul Bulens, Loes Linsen, Jean-Luc Rummens, Geert Robaeys, Eric T de Jonge, Bert Houben, Karin Pat, Daan Walgraeve, Luc Spaas, Jolanda Verheezen, Thessa Verniest, Alexander Goegebuer, Hans Wildiers, Franchette van den Berkmortel, Vivianne C Tjan-Heijnen, Frank Buntinx, Marjan van den Akker

**Affiliations:** 1 Department of General Practice, Katholieke Universiteit Leuven, Kapucijnenvoer 33, bus 7001, 3000 Leuven, Belgium; 2 Department of Medical Oncology, Maastricht University Medical Centre, GROW - School for Oncology and Developmental Biology, P.O. Box 5800, 6202 AZ Maastricht, The Netherlands; 3 Faculty of Medicine, Hasselt University, Campus Diepenbeek, Agoralaan building A, 3590 Diepenbeek, Belgium; 4 Limburgs Oncologisch Centrum, Stadsomvaart 11, 3500 Hasselt, Belgium; 5 Biobank and Clinical Laboratory of Experimental Hematology, Jessa Hospital - campus Virga Jesse, Stadsomvaart 11, 3500 Hasselt, Belgium; 6 Department of Gastroenterology, Ziekenhuis Oost-Limburg, Schiepse Bos 6, 3600 Genk, Belgium; 7 Department of Gynaecology, Ziekenhuis Oost-Limburg, Schiepse Bos 6, 3600 Genk, Belgium; 8 Department of Gastroenterology, Jessa Hospital - campus Virga Jesse, Stadsomvaart 11, 3500 Hasselt, Belgium; 9 Department of Pulmonology, Jessa Hospital - campus Virga Jesse, Stadsomvaart 11, 3500 Hasselt, Belgium; 10 Department of Gastroenterology, Jessa Hospital - campus Salvator, Salvatorstraat 20, 3500 Hasselt, Belgium; 11 Department of Pulmonology, Jessa Hospital - campus Salvator, Salvatorstraat 20, 3500 Hasselt, Belgium; 12 Department of Medical Oncology, Regionaal Ziekenhuis Sint-Trudo, Diestersteenweg 100, 3800 Sint-Truiden, Belgium; 13 Department of Pulmonology, Regionaal Ziekenhuis Heilig Hart Leuven, Naamsestraat 105, 3000 Leuven, Belgium; 14 Department of Gastroenterology, Regionaal Ziekenhuis Heilig Hart Leuven, Naamsestraat 105, 3000 Leuven, Belgium; 15 Department of General Medical Oncology, University Hospitals Leuven, Campus Gasthuisberg, Herestraat 49, 3000 Leuven, Belgium; 16 Department of Internal Medicine, Atrium Medical Centre Parkstad, Henri Dunantstraat 5, P.O. Box 4446, 6401 CX Heerlen, The Netherlands; 17 Department of General Practice, Maastricht University, CAPHRI - School for Public Health and Primary Care, Peter Debeyeplein 1, P.O. Box 616, 6200 MD Maastricht, the Netherlands

## Abstract

**Background:**

Cancer is mainly a disease of older patients. In older cancer patients, additional endpoints such as quality of survival and daily functioning might be considered equally relevant as overall or disease free survival. However, these factors have been understudied using prospective designs focussing on older cancer patients. Therefore, this study will focus on the impact of cancer, ageing, and their interaction on the long-term wellbeing of older cancer patients.

**Methods/Design:**

This study is an observational cohort study. We aim to recruit 720 cancer patients above 70 years with a new diagnosis of breast, prostate, lung or gastrointestinal cancer and two control groups: one control group of 720 patients above 70 years without a previous diagnosis of cancer and one control group of 720 cancer patients between 50 - 69 years newly diagnosed with breast, prostate, lung or gastrointestinal cancer. Data collection will take place at inclusion, after six months, after one year and every subsequent year until death or end of the study. Data will be collected through personal interviews (consisting of socio-demographic information, general health information, a comprehensive geriatric assessment, quality of life, health locus of control and a loneliness scale), a handgrip test, assessment of medical records, two buccal swabs and a blood sample from cancer patients (at baseline). As an annex study, caregivers of the participants will be recruited as well. Data collection for caregivers will consist of a self-administered questionnaire examining depression, coping, and burden.

**Discussion:**

This extensive data collection will increase insight on how wellbeing of older cancer patients is affected by cancer (diagnosis and treatment), ageing, and their interaction. Results may provide new insights, which might contribute to the improvement of care for older cancer patients.

## Background

To a large extent, cancer is a disease of older people [1] and the number of older cancer patients will continue to increase [2]. Older patients have been under-represented in clinical trials [3], which has resulted in a paucity of evidence-based guidelines for treatment of older cancer patients [4]. Over the past decades progress has been made in the field of geriatric oncology, nevertheless gaps remain [5].

One gap is the limited knowledge on the specific impact of cancer diagnosis and treatment on wellbeing or quality of survival of older cancer patients. Nevertheless, "prolongation of active life expectancy" or "quality of survival" is besides prolongation of survival increasingly recognised as an important treatment goal [6].

The impact of cancer diagnosis and treatment highly depends on the age of the patient. The assessment of ageing however is not straightforward. Chronological age by itself is not sufficient to assess ageing [6]. Currently, one's physiological age is best estimated by a comprehensive geriatric assessment (CGA) [7]. A CGA is a multidisciplinary evaluation of an older individual's functional status, comorbidity, cognition, psychological status, social support, nutritional status and review of the patient's medications [8]. Unfortunately, a CGA is very time consuming. Therefore, a two-step approach using screening instruments has been suggested [9]. Examples are the abbreviated comprehensive geriatric assessment (aCGA) [10], the Vulnerable Elders Survey (VES-13) [11], the Groningen Frailty Index (GFI) [12], and the G8 [13] which has been included in the EORTC minimal Dataset [14]. However, the predictive value of these shorter instruments remains to be demonstrated.

Next to the CGA, a person's true age can also be determined by its "molecular/biological" age. From this molecular point of view, telomere length has been described as a measure of ageing as it reflects the organism's age at a cellular level [15-17]. Several studies reported a relationship between telomere length, several age-sensitive measures, and mortality [18-21]. However, more data are required from longitudinal studies that assess the association between telomere length and ageing-related functional measures and the impact of cancer on these parameters.

Age associated changes are also displayed by the immune system, which tends to result in a decreased immune competence also known as "immunosenescence" [22-24]. Currently, several longitudinal studies focusing on the very elderly, have started to reveal immune signatures or biomarkers of immune ageing consisting not of a single parameter, but a cluster of parameters increasingly recognized as an "immune risk profile" or IRP [25-27]. These parameters (CD4/CD8 T cell ratio < 1; cytomegalovirus (CMV) seropositivity; low B cell numbers; poor T cell proliferative responses) might be associated with mortality and aspects of wellbeing in a geriatric population. Therefore, they might potentially be used to identify people at risk for adverse outcomes and consequently develop interventions to delay or postpone these adverse outcomes. However, until now it remains to be determined whether the cluster of immune parameters in the IRP have a predictive value under any other circumstances, for instance in elderly cancer patients. Therefore, we aim to determine whether the IRP and other inflammatory markers (Interleukin-6 (IL-6); Tumour necrosis factor-α (TNF-α); C-reactive protein (CRP)) are associated with wellbeing/mortality in older cancer patients.

Besides patient related characteristics, the social network is a very important factor that influences the wellbeing of older cancer patients. Older cancer patients often rely on the presence of caregivers. Unfortunately, the tasks of caregivers sometimes result in negative consequences to the caregivers' emotional and physical health, which is referred to as caregiver distress [28]. From this perspective, it has been shown that caregivers of older cancer patients are at increased risk for depression, which might result as a consequence of the burden of providing care, but also as a result of inadequate coping mechanisms [29]. Furthermore, depression/distress among caregivers might also negatively affect the patients' wellbeing. Nevertheless, information regarding caregiver distress, burden and coping in relation with older cancer patient characteristics and stage of the illness is still scarce. A long-term follow-up of cancer patients and caregivers will allow us to find prognostic factors for caregiver distress and to give incentives for interventions. Therefore, we aim to determine the impact of patient characteristics (such as change in wellbeing, disease and treatment characteristics) and caregiver characteristics (such as coping strategy and perceived burden) on the occurrence of distress and depression among caregivers.

In conclusion, this study has three major goals: 1) To assess the impact of cancer, ageing and their interaction on subsequent wellbeing of older cancer patients; 2) To determine the association between different measures of ageing (age in life years, a CGA, its screening instruments, telomere length and immunological parameters) and evaluate their ability to predict wellbeing of older cancer patients; 3) To assess the impact of patient and caregiver characteristics on the occurrence of caregivers' distress.

## Methods/Design

### Study design and study population

KLIMOP (Dutch acronym for project on older cancer patients in Belgium and the Netherlands) is a Belgian and Dutch observational prospective cohort study on older cancer patients aged 70 years and above and two control groups; older patients aged 70 years and above without a previous diagnosis of cancer (control for cancer) and cancer patients between 50 - 69 years (control for ageing) (see Table 1). As an annex study, caregivers of participants will be included as well.

**Table 1:** Study design

KLIMOP					
**Cohort - Belgium**			**Cohort - The Netherlands**		
	
**Cases**	**Control group 1**	**Control group 2**	**Cases**	**Control group 1**	**Control group 2**
	
360 Breast, lung and gastro-intestinal cancer patients (≥ 70 years)	360 Patients without a previous diagnosis of cancer (≥ 70 years)	360 Breast, lung and gastro-intestinal cancer patients (50 - 69 years)	360 Breast, lung-, gastro-intestinal and prostate cancer patients (≥ 70 years)	360 Patients without a previous diagnosis of cancer (≥ 70 years)	360 Breast, lung, gastro-intestinal and prostate cancer patients (50 - 69 years)


180 Caregivers	180 Caregivers	180 Caregivers	180 Caregivers	180 Caregivers	180 Caregivers

The study population will consist of Belgian and Dutch participants:

- Cases: 720 older persons (≥ 70 years), with a primary diagnosis of breast, prostate, lung or gastrointestinal cancer. Due to different procedures in the Belgian and Dutch hospitals, prostate cancer patients will not be included in Belgium.

- Control group 1 (control for cancer): 720 older persons (≥ 70 years), without a previous diagnosis of invasive cancer (except non-melanoma cancer of the skin).

- Control group 2 (control for ageing): 720 persons between 50 and 69 years old, with a primary diagnosis of breast, prostate, lung or gastrointestinal cancer. Prostate cancer patients will not be included in Belgium.

The caregiver population will consist of the (potential) caregivers of participating patients. In this study a caregiver is defined as the person who (most often) cares for a needy participant in his or her direct environment, or is most likely to do so when a participant becomes needy. The caregiver is related (family, friends, neighbours, volunteer, ...) to the participant and is not a professional caregiver [30].

Details on the in- and exclusion criteria are listed in Table 2. Control patients who become cases (older patients who become cancer patients) will be considered control and case for the respective periods. Controls, aged 70 years or older who will be diagnosed with cancer, other than breast, prostate, lung or gastrointestinal cancer will drop out. If the patient indicates that there is no caregiver, this will be recorded. If the patient indicates at the first follow-up visit he/she has a caregiver, the caregiver will be included at the first follow-up visit. If the caregiver changes during the study, patients will be asked to indicate the reason. No other caregiver will be recruited.

**Table 2:** In- and exclusion criteria

	Cases:	Control group 1:	Control group 2:	Caregivers
	Cancer patients ≥ 70 years	Patients without cancer ≥ 70 years	Cancer patients 50 - 69 years	
**Inclusion criteria**				

Consenting after being informed	√	√	√	√
Aged 70 years and older	√	√		
Aged between 50 and 69 years			√	
Life expectancy more than 6 months ^1^	√	√	√	
Persons who have a thorough command of Dutch	√	√	√	
Interview within three months after cancer diagnosis	√		√	

**Exclusion criteria**				

Persons with a formal diagnosis of dementia	√	√	√	
Persons with a previous diagnosis of invasive cancer ^2^	√	√	√	
Persons too ill to participate ^1^	√	√	√	

^1 ^based on the judgment of the attending doctor

^2 ^except non-melanoma cancer of the skin

### Recruitment

Up to date, cancer patients are recruited through five hospitals in Belgium (Jessa Ziekenhuis, Ziekenhuis Oost-Limburg, Regionaal Ziekenhuis Sint-Trudo, Regionaal Ziekenhuis Heilig Hart Leuven, Universitair Ziekenhuis Leuven) and two in the Netherlands (Maastricht University Medical Centre and Atrium Medical Centre Parkstad Heerlen). Older patients without cancer are recruited through general practitioners affiliated with the department of General Practice of the K.U.Leuven and Maastricht University Medical Centre.

### Study endpoints and data collection

The primary endpoint of this study is wellbeing of older cancer patients in comparison with younger cancer patients and older patients without a previous diagnosis of cancer. As wellbeing is a very broad concept, we defined four domains that we considered the most important indicators of wellbeing: quality of life, depression, functional status, and comorbidity. The secondary endpoint of this study is caregiver distress. Caregiver distress is defined as the perceived burden and the extent of depressive feelings.

The most important co-variables are ageing, patients' characteristics, and caregivers' characteristics. The different measures of ageing include age in life years, degree of frailty measured by a CGA, the aCGA, the VES-13, the GFI, the G8 and telomere length as a measure of cell ageing. In the future, immunological parameters like Interleukin-6, Tumour necrosis factor-α, C-reactive protein, CD4/CD8 T cell ratio and/or others linked to immunosenescence will be included in a sample of the patients. Patients' characteristics are operationalised as personal characteristics (socio-demographic information, distress, coping strategy, and health locus of control), disease characteristics (type of cancer and TNM classification), and treatment characteristics (type of treatment, treatment completion, complications, hospitalizations and complaints during treatment). Caregivers' characteristics are operationalised by socio-demographic information and coping strategy.

**Figure 1:**
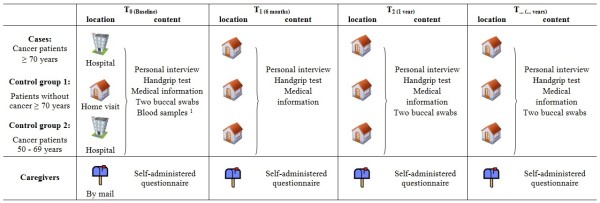
**Study design**. Only at baseline for Belgian cancer patients

Data collection will take place at inclusion, after six months of follow-up, after one year of follow-up and every subsequent year until death or end of the study (see Figure 1). For cancer patients and older patients without cancer, data will be collected through personal interviews (consisting of socio-demographic information, general health information, a comprehensive geriatric assessment, quality of life, health locus of control and a loneliness scale), a handgrip test, medical information (extracted from the medical record) and two buccal swabs. In Belgium, a baseline blood sample will be collected as well for all cancer patients. The baseline interview of cancer patients (≥ 70 years and 50 - 69 years) will take place at the hospital, scheduled together with other appointments. The baseline interview of older patients without cancer will take place during home visits, as well as the follow-up interviews of both cancer and non-cancer patients. For caregivers, data will be collected through self-administered questionnaires, which are partially given to them by the patients and partially sent by mail. A stamped envelope will be enclosed. The content of the data collection is summarized in Table 3.

**Table 3:** Data collection

Setting		Data collection
**Interview**	Ageing: CGA	
	Functional	Activities of Daily Living (ADL): Barthel index [41,42]
		Instrumental Activities of Daily Living (IADL): Lawton IADL scale [43]
		Handgrip strength: Hydraulic Jamar hand-held dynamometer
	Polypharmacy	
	Depression	15-item Geriatric Depression Scale (GDS-15) [44]
	Cognition	Mini Mental State Examination [45]
	Nutritional status	Participants will be asked whether they lost or gained weight in the past three months and how much ^1^
	Social support	Questions considering the availability of a caregiver and professional care

		The loneliness scale [46]
	
	Ageing: Screening instruments	Abbreviated CGA (aCGA) [10,47]
		Vulnerable Elders Survey - 13 (VES-13) [11]
		Groningen Frailty Index (GFI) [12]
		G8 [13] which has been included in the EORTC minimal Dataset [14]
	
	Quality of life	EORTC QLQ-C30 [48]
	
	Distress	Distress Barometer (DB) [49]
	
	Coping strategy	The Utrecht Coping List (UCL) [50]
	
	Opinion on health and disease	Health Locus of control (HLoC) [51]

**Medical record**	Survival	Date of death
	
	Comorbidity	Charlston Comorbidity Index [52] + additional diseases ^2^
	
	Disease characteristics	Data of cancer diagnosis, TNM classification, type of cancer
	
	Treatment characteristics	Type of treatment
		Completion of therapy
		Complications
		Complaints (e.g. fatigue, pain)
		Hospitalizations
	
	Medication	

**Buccal swabs**	Ageing: biological parameters	Telomere length
		
**Baseline blood samples **^**3**^		CD4/CD8 T cell ratio, CMV status, B cell number, IL-6, TNF-α CRP etc.

**Questionnaire caregivers**	Socio-demographic information	
	
	Coping strategy of caregiver	The Utrecht Coping List (UCL) [50]
	
	Caregivers distress	15-item Geriatric Depression scale (GDS15) [44]
		12-item Zarit Burden Inventory (ZBI) [53]

^1 ^We decided that the extra information given by the Mini Nutritional Assessment would not outweigh the burden of 18 extra questions, including circumferences of the calf and upper arm.

^2 ^A limitation of the Charlston Comorbidity Index is that it does not take into account some disorders that might affect prognosis or evolution of quality of life in cancer patients. Therefore, some additional diseases will be registered as well (parkinsonism, blood transfusions, transplantations, thromboses, lung embolisms, a pacemaker, angina pectoris, a PCTA or a coronary bypass).

^3 ^Only for cancer patients in Belgium

### Sample size calculation

Sample size calculations were based on the primary objective of this study: to determine the impact of cancer diagnosis, ageing, and their interaction on subsequent wellbeing of older cancer patients. Separate instruments such as the GDS-15 measure the four domains that we considered the most important indicators of wellbeing. Therefore, the sample size was calculated for depression as a dichotomous endpoint. The prevalence of depression has been reported to be about 25% in older cancer patients [31], while the prevalence of depression among community-dwelling older non-cancer patients is considerably lower (<15%) [32]. However, higher and lower prevalences of depression have been reported as well. Therefore, we simulated a few sample size calculations with:

- differing prevalences of depression in the control group: ranging from 25% to 65%

- a relative risk for disease (depression) in older cancer patients relative to controls of 1.5

- a power to reject the null hypothesis of 80%

- a type I error probability of 0.05

- a ratio of cases versus controls of one

Assuming these specifications, the total needed sample size was estimated between 626 patients (assuming a prevalence of depression of 25% in the control group) and 100 (assuming a prevalence of depression of 65% in the control group). In comparison with other studies on older patients, we assumed a loss to follow-up and a yearly mortality rate of 10% among the two control groups and a loss-to follow-up and a yearly mortality rate of 20% among older cancer patients [33]. Finally, per country, a sample size of 360 cases (cancer patients aged 70 years and older), 360 controls (group 1: cancer patients between 50 - 69 years) and 360 controls (group 2: older patients without a previous diagnosis of cancer) per country was proposed, enabling within-country analyses.

For caregivers, sample size was also calculated for depression as endpoint (instead of the combination of depression and burden). The prevalence of depression among caregivers ranges between 32 - 50% [34]. Applying the same assumptions as for older cancer patients (relative risk of 1.5, a prevalence of depression of 25% - 55% in the control group, a power of 80% and α 0.05) the estimated sample size was similar (626 - 100). As some patients will not have a caregiver, we expect it feasible to recruit about half of the caregivers of all participants. We expect to recruit about 550 caregivers per country.

### Ethics

The study protocol was approved by the Ethical Committee of the K.U.Leuven and U.Z.Leuven (S52097 - ML6279) (Belgium) and by the Medical Ethics Committee of the Maastricht University Medical Centre (NL31414.068.10) (the Netherlands). The study will be conducted in compliance with Good Clinical Practice guidelines Procedures (GCP), the principles of the Declaration of Helsinki (version October 2008) and the Belgian (law of 7 may 2004 concerning clinical trials with humans) and Dutch (Medical Research Involving Human Subjects Act and Personal Data Protection Act) laws.

### Data check and analysis

All interviews and other data collection (e.g. medical information) are processed using software developed for this project. Data is entered directly in the database using this program. The quality of the data entry is verified by automated checking for erroneous or missing entries. Standard statistical analyses will be used for describing patient characteristics and comparing older cancer patients with the two control groups at baseline and during follow-up. When applicable more complex statistical methods such as multivariable logistic regression, survival analysis and methods to deal with repeated measurements will be used. In all analyses, two sided p-values will be used at a significance level of 0.05. In all future publications we will follow the STROBE criteria (Strengthening The Reporting of OBservational studies in Epidemiology) [35].

## Discussion

To our knowledge, this is the first prospective cohort study that focuses on the wellbeing of older long-term cancer survivors. This is surprising as the number of older cancer patients is expected to increase dramatically due to the ageing of the population and advances in early detection and cancer treatments [2].

It has been repeatedly shown that older cancer patients are "less willing to compromise their quality of life for the potential of increased survival" [36]. Hence, in the geriatric oncology setting, traditional endpoints as survival might be less relevant. As suggested by Hurria & Balducci, other endpoints such as the quality of survival may be considered equally relevant [37]. However, information on the quality of survival is lacking. Previous prospective studies that focused at aspects of quality of survival such as functional status and quality of life had only limited follow-up. For example, three prospective studies of Puts et al., Minisine et al., and Marinello et al. investigated the functional status in older cancer patients, but only until 6 months of follow-up [38-40]. Therefore, this study is unique as we aim to evaluate the quality of survival (here defined as wellbeing) of older cancer patients for an extended period. This study will provide insight in the evolution of wellbeing (in terms of Qol, depression, functional status and comorbidity) and the most important determinants among older cancer survivors in comparison with younger cancer survivors and older patients without a previous diagnosis of cancer. We expect to find associations between different measures of ageing and wellbeing of older cancer patients, which could help us identify people that need interventions to assure quality of survival and to tailor treatment for older cancer patients.

## Abbreviations

aCGA: Abbreviated comprehensive geriatric assessment; ADL: Activities of daily living; CGA: Comprehensive geriatric assessment; CMV: cytomegalovirus; CRP: C reactive protein; DB: Distress barometer; EORTC Elderly MinsDS: EORCT Elderly Minimal Dataset; GDS-15: Geriatric depression scale; GFI: Groningen Frailty Index; HLoC: Health locus of control; IADL: Instrumental activities of daily living; IL-6: Interleukin 6; IRP: Immune Risk Profile; KLIMOP: Dutch acronym for project on older cancer patients in Belgium and the Netherlands; MMSE: Mini mental state examination; STROBE: Strengthening the reporting of observational studies in epidemiology; TNF-α: Tumour necrosis factor α; UCL: The Utrecht Coping List; VES-13: Vulnerable Elders Survey; ZBI: Zarit Burden Interview

## Competing interests

The authors declare that they have no competing interests.

## Authors' contributions

LDe drafted the manuscript and contributes to the data collection. DvA, LDa, and KN contribute to the data collection. PS participated in the coordination of the study. PB contributed to the conception of the study and participated in the design and coordination of the study. LL and JLR contribute to the storage and processing of blood samples and buccal swabs. GR, ETdJ, BH, KP, DW, LS, JV, TV, AG, and HW coordinate the recruitment of cancer patients in the respective departments. FvdB and VCTH contributed to the design and the coordination of the study. FB contributed to the conception, the design, and coordination of the study. MvdA participated in the design and coordination of the study. All authors agreed with the final version of the manuscript and critically revised the manuscript.

## Pre-publication history

The pre-publication history for this paper can be accessed here:

http://www.biomedcentral.com/1471-2458/11/825/prepub
